# How can we promote vaccination of the mass population?—Lessons from the COVID-19 vaccination defaults

**DOI:** 10.1371/journal.pone.0298983

**Published:** 2024-02-16

**Authors:** Masaki Takebayashi, Mira Namba, Yudai Kaneda, Tatsuya Koyama, Soichiro Miyashita, Kurenai Takebayashi, Motoki Ohnishi

**Affiliations:** 1 Graduate School of Health Sciences, Aomori University of Health and Welfare, Aomori, Japan; 2 Faculty of Sociology, Aomori University, Aomori, Japan; 3 School of Medicine, Keio University, Tokyo, Japan; 4 School of Medicine, Hokkaido University, Sapporo, Hokkaido, Japan; 5 Faculty of Human Life Sciences, Mimasaka University, Tsuyama, Okayama, Japan; 6 Aomori Prefectural Government, Aomori, Japan; 7 Olympus Corporation, Tokyo, Japan; Lahore Medical and Dental College, PAKISTAN

## Abstract

While vaccines are pivotal in combating COVID-19, concerns about side effects and complex procedures have hindered complete vaccination. Prior studies suggest that individuals defaulted to opt-out exhibit higher COVID-19 vaccination rates compared to those in opt-in systems. However, these studies were conducted in countries with a tolerant attitude towards vaccination and default changes, targeting specific age groups, and did not address potential deterrents like the increase in cancellation rates on the day, discomfort towards changing defaults, or the possibility of the opt-out effect being a one-time occurrence. Under the hypothesis that the default nature of the COVID-19 vaccination system influences attitudes towards vaccination even in countries conservative about vaccination and default changes like in Japan, we aimed to examine the differences in the first and second dose vaccination rates, cancellation rates, and the number of complaints between the opt-in and opt-out systems for COVID-19 vaccination. An email survey was conducted in 10 cities in A Prefecture, Japan. The results showed not only higher COVID-19 vaccination rates across all comparable age groups in the opt-out group but also a notably smaller decrease in the second-dose vaccination rate compared to the opt-in group, all achieved without any complaints about the system’s introduction. Consequently, it can be inferred that the potential inhibiting factors were largely overcome. Despite some limitations, such as regional specificity, the study suggests that opt-out systems might increase COVID-19 vaccination coverage without leading to significant cancellations or complaints, presenting a promising strategy to facilitate vaccination efforts.

## I Introduction

Vaccination is the key infection control measure against Coronavirus Disease 2019 (COVID-19) [1]. However, not all targeted individuals have undergone vaccination, with some refraining due to apprehensions about side effects, while others are deterred by the intricacies in procedural complexities, even though they might be willing to receive the vaccine [2, 3]. A systematic review on COVID-19 vaccine promotion reported several methods, but very few measured real behavioral outcomes [4]. Among the interventions tested in real-world settings, one of the most effective was changing the default nudge, defined as the pre-selected option automatically applied when the chooser takes no active decision [5–9]. Indeed, individuals with an opt-out default (initially scheduled for vaccination, requiring contact only if inconvenient) exhibited a 32% relative increase in vaccination rates (13.1% vs. 9.9%) compared to those in the opt-in system (where participants adjust and schedule their vaccination) [5]. However, this study was conducted in Italy, a country more tolerant of default changes and without significant vaccine hesitancy in international comparison and focusing on a target age group of 50–59 years [5, 10, 11]. Thanks to the favorable conditions, the results may have been predisposed to positive results. For the opt-out approach in COVID-19 vaccination to gain wider acceptance, data from countries with lower vaccine acceptance and resistance to default changes, encompassing a broader age range, would be preferable, as is typical in countries like Japan [11, 12]. Additionally, the study did not address potential inhibiting factors associated with the implementation of the opt-out system, such as increased cancellation rates on the day, discomfort among individuals regarding the change in defaults, or the possibility that the opt-out effect may be a one-time occurrence [5, 13–15].

In this study, under the hypothesis that differences in the default options for COVID-19 vaccination systems influence vaccination attitudes even in countries conservative about vaccination and default changes, we aimed to examine the differences in first and second dose vaccination rates, cancellation rates, and the number of complaints between the opt-in and opt-out systems for COVID-19 vaccination.

## II Methods

### 1 Study design

Observational study.

### 2 Target

Generally, local municipalities in Japan are responsible for providing vaccination information services to residents, and most have adopted the opt-in system for COVID-19 vaccination [16]. This study’s target encompassed all 10 cities in A Prefecture, Japan. While there are 30 other municipalities in A Prefecture, their attitudes toward vaccination are not standardized, with some smaller towns and villages having administrative staff directly explain vaccination to residents. In contrast, cities primarily implement vaccination recommendations through notifications sent to residents and are less likely to experience such variations; hence, to minimize the impact of other factors, only city municipalities were included in this study.

The preliminary survey confirmed that 9 out of 10 cities had adopted opt-in and only city B had adopted a hybrid opt-in/opt-out system; the opt-in system was designated for those with an urgent need, such as seniors 65 years of age and older, or 12-14-year-olds required careful parental judgment; otherwise, the opt-out system was designated.

### 3 Survey

Vaccination status such as vaccination rates and cancellation rates as of 10 October 2021 of all the 10 cities in A Prefecture were surveyed. We set 10 October 2021 as the record date because the Japanese Government has announced that 90% of the population aged 12 and over could complete the two-dose vaccination scheme by this date [17]. While most of this information was usually publicly accessible, the formats varied by city, and some older data were inaccessible, necessitating email inquiries.

Primary outcome was the first and second vaccination coverage by age group (12 years and older). Secondary outcome was the cancellation rate on the day of vaccination (i.e., the percentage of people who made an appointment but did not show up at the vaccination sites on the day of vaccination without informing their cancellation by the day before). In addition, municipalities with opt-out systems were also asked about the number of complaints they received about their system.

### 4 Statistical analysis

Cities other than City B were considered an opt-in group, and those eligible for opt-out in City B were considered an opt-out group. Vaccination rates for the comparable age group were examined across different groups, and for each municipality’s vaccination rate, a 95% confidence interval was calculated and analyzed descriptively. Cancellation rates were compared between municipalities that had responded to our inquiries. IBM SPSS Statistics 24 (IBM Japan, Tokyo, Japan) was used for analysis.

### 5 Ethics statement

Ethical approval was deemed not applicable for this study since this was a non-human subject research and only publicly available data were used. Prior confirmation of this was obtained from the ethics review committee at the first author’s affiliated university. All data were fully anonymized before we accessed them.

## III Results

All ten cities responded, and one city reported not knowing the vaccination coverage, so the remaining nine cities were included in the analysis. Table 1 shows the vaccination coverage in each city.

**Table 1:** Vaccination coverage rate.

City	System	Dose		20–64 age		
					95% Confidence Interval	
					Minimum	Maximum
B	Opt-out	1	Nominated Population	14,903	88.2%	89.2%
			Vaccinated Population	13,215		
			Vaccination Coverage	88.7%		
		2	Nominated Population	14,903	84.9%	86.0%
			Vaccinated Population	12,732		
			Vaccination Coverage	85.4%		
C	Opt-in	1	Nominated Population	137,471	80.3%	80.7%
			Vaccinated Population	110,670		
			Vaccination Coverage	80.5%		
		2	Nominated Population	137,471	68.0%	68.5%
			Vaccinated Population	93,857		
			Vaccination Coverage	68.3%		
D	Opt-in	1	Nominated Population	118,922	75.0%	75.5%
			Vaccinated Population	89,470		
			Vaccination Coverage	75.2%		
		2	Nominated Population	118,922	51.3%	51.9%
			Vaccinated Population	61,385		
			Vaccination Coverage	51.6%		
E	Opt-in	1	Nominated Population	87,354	72.7%	73.3%
			Vaccinated Population	63,791		
			Vaccination Coverage	73.0%		
		2	Nominated Population	87,354	54.9%	55.5%
			Vaccinated Population	48,219		
			Vaccination Coverage	55.2%		
F	Opt-in	1	Nominated Population	30,165	82.8%	83.6%
			Vaccinated Population	25,102		
			Vaccination Coverage	83.2%		
		2	Nominated Population	30,165	71.6%	72.6%
			Vaccinated Population	21,736		
			Vaccination Coverage	72.1%		
G	Opt-in	1	Nominated Population	26,743	76.6%	77.6%
			Vaccinated Population	20,623		
			Vaccination Coverage	77.1%		
		2	Nominated Population	26,743	58.3%	59.5%
			Vaccinated Population	15,751		
			Vaccination Coverage	58.9%		
H	Opt-in	1	Nominated Population	21,549	78.3%	79.4%
			Vaccinated Population	16,988		
			Vaccination Coverage	78.8%		
		2	Nominated Population	21,549	66.7%	68.0%
			Vaccinated Population	14,510		
			Vaccination Coverage	67.3%		
I	Opt-in	1	Nominated Population	14,582	75.5%	76.9%
			Vaccinated Population	11,111		
			Vaccination Coverage	76.2%		
		2	Nominated Population	14,582	57.2%	58.8%
			Vaccinated Population	8,463		
			Vaccination Coverage	58.0%		
J	Opt-in	1	Nominated Population	15,463	72.0%	73.4%
			Vaccinated Population	11,247		
			Vaccination Coverage	72.7%		
		2	Nominated Population	15,463	60.4%	61.9%
			Vaccinated Population	9,457		
			Vaccination Coverage	61.2%		

The opt-in group had a 95% confidence interval of 74.1% - 80.1% for the first and 55.7%–67.5% for the second dose.

The opt-out group had a 95% confidence interval of 88.2%-89.2% for the first and 84.9%-86.0% for the second dose, compared to 74.1%–80.1% for the first and 55.7%–67.5% for the second dose for the opt-in group, both of which were higher in the opt-out group. Specifically, the data showed that in the opt-in group, there was a decrease of 11.1–23.6 percentage points in the vaccination rate for the second dose compared to the first, whereas the opt-out group experienced a much smaller reduction, limited to just 3.2 percentage points.

Table 2 shows the no-show and cancellation rate on the day in City B, as other cities did not record such information. The data shows that for the first dose, the vaccination rates in the opt-out group, the rates varied from 7.3% to 17.7% averaging 11.3% overall. For the second dose, the opt-out group showed an increase, ranging from 9.4% to 22.6%, with an overall average of 14.6%.

**Table 2:** No-show and cancellation rate on the day of the vaccination.

	TOTAL	12–14	15–19	20–29	30–39	40–49	50–59	60–64	65–69	70–79	80–89	90–99	100 AND MORE
**1ST OPT-IN**	9.7%	14.6%	19.1%	18.8%	12.3%	10.3%	7.9%	6.7%	7.6%	7.2%	8.8%	11.3%	10.0%
**1ST OPT-OUT**	11.3%	–	11.5%	17.7%	13.5%	10.8%	8.9%	7.3%	–	–	–	–	–
**2ND OPT-IN**	10.5%	15.6%	21.5%	20.0%	13.5%	11.4%	9.0%	7.1%	8.0%	7.8%	9.3%	12.3%	12.5%
**2ND OPT-OUT**	14.6%	–	14.8%	22.6%	17.1%	14.0%	11.6%	9.4%	–	–	–	–	–

^※^As stated earlier, since opt-in and opt-out subjects in City B have very different basic characteristics, we did not perform a statistical analysis of the difference between the two.

Also, in the opt-out group, no complaints were received from residents about the implementation of the opt-out system.

## IV Discussion

Our study revealed that in all comparable age groups between 20 and 64 years, COVID-19 vaccination rates were higher in the opt-out group than in the opt-in group, as indicated within the 95% confidence interval. This finding supports our hypothesis that "even in countries conservative about vaccination and default changes, differences in the default options for COVID-19 vaccination can influence vaccination attitudes." This aligns with meta-analyses suggesting that the default nudge is reported to have the most substantial influence on altering people’s behavior [18, 19]. Our study implies that the opt-out approach for COVID-19 vaccination might be effective across different cultural contexts and age groups, suggesting its potential applicability in countries like Pakistan, where vaccine hesitancy is prevalent [12, 20], or in other nudge-cautious countries like Hungary [11]. Thus, in considering its practical application, it is essential to contemplate the inhibiting factors identified.

Cancellation rates were unavailable except for City B, so comparisons between groups could not be conducted. However, the cancellation rate in the opt-out group for COVID-19 was not as high as that for the influenza vaccine of reported 52% in opt-out settings, indicating potentially positive data for implementing opt-out strategies [21]. In opt-in systems, the burden of operation is concentrated on vaccination personnel due to simultaneous handling of appointment receptions, progress management, and cancellation management. In contrast, opt-out systems allow securing vaccination dates and locations in advance, enabling focused management of progress and cancellations thereafter. The insights gained from the cancellation rates in the opt-out group in this study suggest the feasibility of creating waiting lists for cancellations.

In addition, City B did not receive any complaints about implementing an opt-out system, suggesting that even among the Japanese, who are generally cautious about changes in default settings, the opt-out system was accepted without significant dissatisfaction. Opt-out approaches appeal to addressing behavioral inertia, especially when individuals face psychological barriers such as limited capacity to evaluate and compare choice options [19]. In the opt-out group of this study, it is inferred that the discomfort was reduced as individuals were relieved from the process of enrollment. On the other hand, in opt-in systems, there have been instances, such as servers crashing due to a surge in COVID-19 vaccine applications, leading to resident dissatisfaction, suggesting that the opt-in group might have experienced more complaints [22].

Furthermore, the opt-out group experienced a more limited decrease in vaccination rates from the first to the second dose compared to the opt-in group. Systematic reviews indicate that nudging may be effective in producing immediate behavioral changes; however, there is little evidence that nudging interventions result in lasting behavioral changes [23]. Nevertheless, in the context of COVID-19 vaccination, it appears that the influence may last for at least two doses. This can be explained by the fact that opt-out strategies powerfully address behavioral inertia [19]. Conversely, in the opt-in group, dissatisfaction experienced during the application for the first dose might have contributed to a lower uptake of the second dose.

Consequently, we can infer that the potential inhibiting factors related to the opt-out approach have been largely cleared. This study not only demonstrated increased vaccination rates but also indicated that the inhibiting factors were minimal, suggesting benefits for both the vaccine recipients and administrators. Although the effectiveness of default nudges in increasing vaccination rates has been recognized internationally, Japan has shown conservative responses in handling the COVID-19 pandemic, with adherence to initial methods, and thus, such approaches were not readily implemented [24]. Based on the findings of this study, it is considered advisable to include the option of implementing an opt-out system in the strategies for future pandemics beyond COVID-19.

This study has several limitations. First, the scale of infection rates varies from city to city; thus, it cannot be ruled out that this may impact vaccination coverage. As the number of outbreaks or infected cases by the city was not available, their use in the analysis was not feasible. Secondly, since this study was conducted in only one region and only one city adopted the opt-out system, there are still points to be clarified before these results can be immediately generalized. Specifically, particular factors within City B (such as the strength of the mayor’s leadership) may have influenced the vaccination rate. However, this study was unable to examine these factors. Further research, including qualitative research, is needed to overcome these limitations. Despite these limitations, this study is a substantive inquiry suggesting that the opt-out approach to COVID-19 vaccination could lead to higher vaccination rates, without being significantly hindered by factors like high cancellation rates and unpopularity.

In conclusion, our study implies that the opt-out approach to COVID-19 vaccination in Japan, characterized by strong vaccine hesitancy and conservative attitudes towards default changes, increased vaccination rates across 20–64 age groups, overcoming potential inhibiting factors such as higher cancellation rates, unpopularity, and limited effectiveness of the intervention. These results underline the potential of behavioral interventions like the opt-out system in enhancing public health responses, especially in the context of potential pandemics. However, given the study’s unique factors within the single city that adopted the opt-out system, further research is warranted to validate these findings and explore their broader applicability.

## Supporting information

S1 File Details of vaccination status in each city. (DOCX)
