# Supplementary material for: How can we promote vaccination of the mass population?—Lessons from the COVID-19 vaccination defaults
Source: PLoS One. 2024 Feb 16;19(2):e0298983. doi: 10.1371/journal.pone.0298983 (PMC10871499; doi:10.1371/journal.pone.0298983)
Supplement: S1 File — (DOCX) [file pone.0298983.s001.docx]

Details of vaccination status in each city

| City | System | Total | | Vaccination coverage by age group (%) | | | | | | | | | | | |
| --- | --- | --- | --- | --- | --- | --- | --- | --- | --- | --- | --- | --- | --- | --- | --- |
|  |  |  |  | 12-14 | 15-19 | 20-29 | 30-39 | 40-49 | 50-59 | 60-64 | 65-69 | 70-79 | 80-89 | 90-99 | 100- |
| B | Opt-in | Nominated Population | 34,523 | 1,294 | 1,067 | 2,244 | 2,569 | 3,360 | 3,353 | 1,880 | 4,588 | 8,318 | 4,660 | 1,150 | 40 |
|  |  | Vaccinated Population | 31,162 | 1,105 | 863 | 1,823 | 2,253 | 3,014 | 3,088 | 1,754 | 4,239 | 7,718 | 4,249 | 1,020 | 36 |
|  |  | Vaccination Coverage | 90.3% | 85.4% | 80.9% | 81.2% | 87.7% | 89.7% | 92.1% | 93.3% | 92.4% | 92.8% | 91.2% | 88.7% | 90.0% |
|  |  | Nominated Population | 34,523 | 1,294 | 1,067 | 2,244 | 2,569 | 3,360 | 3,353 | 1,880 | 4,588 | 8,318 | 4,660 | 1,150 | 40 |
|  |  | Vaccinated Population | 30,887 | 1,092 | 838 | 1,795 | 2,223 | 2,978 | 3,050 | 1,746 | 4,220 | 7,673 | 4,228 | 1,009 | 35 |
|  |  | Vaccination Coverage | 89.5% | 84.4% | 78.5% | 80.0% | 86.5% | 88.6% | 91.0% | 92.9% | 92.0% | 92.2% | 90.7% | 87.7% | 87.5% |
| B | Opt-out | Nominated Population | 16,225 | 0 | 1,322 | 2,150 | 2,755 | 4,111 | 3,867 | 2,020 | 0 | 0 | 0 | 0 | 0 |
|  |  | Vaccinated Population | 14,385 | 0 | 1,170 | 1,770 | 2,383 | 3,666 | 3,524 | 1,872 | 0 | 0 | 0 | 0 | 0 |
|  |  | Vaccination Coverage | 90.3% | ー | 88.5% | 82.3% | 86.5% | 89.2% | 91.1% | 92.7% | ー | ー | ー | ー | ー |
|  |  | Nominated Population | 16,225 | 0 | 1,322 | 2,150 | 2,755 | 4,111 | 3,867 | 2,020 | 0 | 0 | 0 | 0 | 0 |
|  |  | Vaccinated Population | 13,858 | 0 | 1,126 | 1,664 | 2,284 | 3,536 | 3,418 | 1,830 | 0 | 0 | 0 | 0 | 0 |
|  |  | Vaccination Coverage | 85.4% | ー | 85.2% | 77.4% | 82.9% | 86.0% | 88.4% | 90.6% | ー | ー | ー | ー | ー |
| C | Opt-in | Nominated Population | 255,534 | 30,632 | | 23,522 | 15,492 | 39,655 | 38,742 | 20,060 | 87,431 | | | | |
|  |  | Vaccinated Population | 214,700 | 21,929 | | 17,136 | 11,602 | 31,530 | 32,884 | 17,518 | 81,984 | | | | |
|  |  | Vaccination Coverage | 84.0% | 71.6% | | 72.9% | 74.9% | 79.5% | 84.9% | 87.3% | 93.8% | | | | |
|  |  | Nominated Population | 255,534 | 30,632 | | 23,522 | 15,492 | 39,655 | 38,742 | 20,060 | 87,431 | | | | |
|  |  | Vaccinated Population | 190,552 | 16,033 | | 13,431 | 9,174 | 25,994 | 28,874 | 16,383 | 80,611 | | | | |
|  |  | Vaccination Coverage | 74.6% | 52.3% | | 57.1% | 59.2% | 65.6% | 74.5% | 81.7% | 92.2% | | | | |
| D | Opt-in | Nominated Population | 206,481 | 17,624 | | 18,480 | 22,742 | 31,072 | 31,377 | 15,251 | 69,935 | | | | |
|  |  | Vaccinated Population | 165,061 | 11,746 | | 12,777 | 16,001 | 23,251 | 24,819 | 12,622 | 63,172 | | | | |
|  |  | Vaccination Coverage | 79.9% | 66.7% | | 69.1% | 70.4% | 74.8% | 79.1% | 82.8% | 90.3% | | | | |
|  |  | Nominated Population | 206,481 | 17,624 | | 18,480 | 22,742 | 31,072 | 31,377 | 15,251 | 69,935 | | | | |
|  |  | Vaccinated Population | 129,897 | 4,558 | | 7,871 | 10,143 | 15,390 | 18,126 | 9,855 | 62,263 | | | | |
|  |  | Vaccination Coverage | 62.9% | 25.9% | | 42.6% | 44.6% | 49.5% | 57.8% | 64.6% | 89.0% | | | | |
| E | Opt-in | Nominated Population | 153,075 | 3,820 | 7,284 | 14,281 | 16,535 | 21,937 | 22,756 | 11,845 | 12,316 | 23,897 | 14,494 | 3,793 | 117 |
|  |  | Vaccinated Population | 119,858 | 1,780 | 4,443 | 9,289 | 10,848 | 15,829 | 17,825 | 10,000 | 11,117 | 21,842 | 13,356 | 3,499 | 101 |
|  |  | Vaccination Coverage | 78.3% | 46.6% | 61.0% | 65.0% | 65.6% | 72.2% | 78.3% | 84.4% | 90.3% | 91.4% | 92.1% | 92.2% | 86.3% |
|  |  | Nominated Population | 153,075 | 3,820 | 7,284 | 14,281 | 16,535 | 21,937 | 22,756 | 11,845 | 12,316 | 23,897 | 14,494 | 3,793 | 117 |
|  |  | Vaccinated Population | 99,856 | 596 | 2,475 | 6,688 | 7,539 | 11,411 | 14,075 | 8,506 | 10,688 | 21,296 | 13,082 | 3,401 | 99 |
|  |  | Vaccination Coverage | 65.2% | 15.6% | 34.0% | 46.8% | 45.6% | 52.0% | 61.9% | 71.8% | 86.8% | 89.1% | 90.3% | 89.7% | 84.6% |
| F | Opt-in | Nominated Population | 55,416 | 1,417 | 2,610 | 4,605 | 5,271 | 7,952 | 8,020 | 4,317 | 4,818 | 9,331 | 5,489 | 1,524 | 62 |
|  |  | Vaccinated Population | 47,048 | 349 | 2,028 | 3,366 | 4,074 | 6,704 | 7,034 | 3,924 | 4,466 | 8,687 | 5,017 | 1,343 | 55 |
|  |  | Vaccination Coverage | 84.9% | 24.6% | 77.7% | 73.1% | 77.3% | 84.3% | 87.7% | 90.9% | 92.7% | 93.1% | 91.4% | 88.1% | 88.5% |
|  |  | Nominated Population | 55,416 | 1,417 | 2,610 | 4,605 | 5,271 | 7,952 | 8,020 | 4,317 | 4,818 | 9,331 | 5,489 | 1,524 | 62 |
|  |  | Vaccinated Population | 42,172 | 9 | 1,156 | 2,593 | 3,210 | 5,733 | 6,440 | 3,760 | 4,404 | 8,603 | 4,946 | 1,317 | 50 |
|  |  | Vaccination Coverage | 76.1% | 0.6% | 44.3% | 56.3% | 60.9% | 72.1% | 80.3% | 87.1% | 91.4% | 92.2% | 90.1% | 86.4% | 80.6% |
| G | Opt-in | Nominated Population | 48,811 | 1128 | 2159 | 3683 | 4724 | 6514 | 7619 | 4203 | 4414 | 7724 | 5268 | 1339 | 36 |
|  |  | Vaccinated Population | 40,390 | 937 | 1355 | 2131 | 2753 | 5346 | 6614 | 3779 | 4066 | 7161 | 4933 | 1279 | 36 |
|  |  | Vaccination Coverage | 82.7% | 83.1% | 62.8% | 57.9% | 58.3% | 82.1% | 86.8% | 89.9% | 92.1% | 92.7% | 93.6% | 95.5% | 100.0% |
|  |  | Nominated Population | 48,811 | 1128 | 2159 | 3683 | 4724 | 6514 | 7619 | 4203 | 4414 | 7724 | 5268 | 1339 | 36 |
|  |  | Vaccinated Population | 34,713 | 878 | 801 | 1553 | 1902 | 3024 | 5842 | 3430 | 4039 | 7099 | 4862 | 1249 | 34 |
|  |  | Vaccination Coverage | 71.1% | 77.8% | 37.1% | 42.2% | 40.3% | 46.4% | 76.7% | 81.6% | 91.5% | 91.9% | 92.3% | 93.3% | 94.4% |
| H | Opt-in | Nominated Population | 39,323 | 1,099 | 1,826 | 4,043 | 4,722 | 5,344 | 5,009 | 2,431 | 2,645 | 4,299 | 2,664 | 748 | 16 |
|  |  | Vaccinated Population | 28,902 | 393 | 1,337 | 2,949 | 3,460 | 4,208 | 4,286 | 2,084 | 2,320 | 4,247 | 2,674 | 904 | 39 |
|  |  | Vaccination Coverage | 73.5% | 35.8% | 73.2% | 72.9% | 73.3% | 78.7% | 85.6% | 85.7% | 87.7% | 98.8% | 100.4% | 120.9% | 245.8% |
|  |  | Nominated Population | 39,323 | 1,099 | 1,826 | 4,043 | 4,722 | 5,344 | 5,009 | 2,431 | 2,645 | 4,299 | 2,664 | 748 | 16 |
|  |  | Vaccinated Population | 25,167 | 79 | 511 | 2,123 | 2,753 | 3,618 | 4,011 | 2,005 | 2,281 | 4,208 | 2,635 | 904 | 39 |
|  |  | Vaccination Coverage | 64.0% | 7.2% | 28.0% | 52.5% | 58.3% | 67.7% | 80.1% | 82.5% | 86.2% | 97.9% | 98.9% | 120.9% | 245.8% |
| I | Opt-in | Nominated Population | 28,932 | 1,075 | 1,161 | 1,888 | 2,729 | 3,536 | 3,943 | 2,486 | 2,712 | 4,814 | 3,462 | 977 | 17 |
|  |  | Vaccinated Population | 23,319 | 189 | 714 | 1,214 | 1,595 | 2,779 | 3,344 | 2,179 | 2,499 | 4,478 | 3,315 | 993 | 20 |
|  |  | Vaccination Coverage | 80.6% | 17.6% | 61.5% | 64.3% | 58.4% | 78.6% | 84.8% | 87.7% | 92.1% | 93.0% | 95.8% | 101.6% | 100.0% |
|  |  | Nominated Population | 28,932 | 1,075 | 1,161 | 1,888 | 2,729 | 3,536 | 3,943 | 2,486 | 2,712 | 4,814 | 3,462 | 977 | 17 |
|  |  | Vaccinated Population | 19,966 | 10 | 361 | 802 | 1,005 | 2,238 | 2,510 | 1,908 | 2,463 | 4,422 | 3,268 | 961 | 20 |
|  |  | Vaccination Coverage | 69.0% | 0.9% | 31.1% | 42.5% | 36.8% | 63.3% | 63.7% | 76.7% | 90.8% | 91.9% | 94.4% | 98.4% | 100.0% |
| J | Opt-in | Nominated Population | 28,028 | 1,989 | | 2,234 | 3,044 | 3,924 | 4,005 | 2,256 | 2,569 | 2,648 | 5,351 | | |
|  |  | Vaccinated Population | 22,422 | 1,386 | | 1,278 | 1,997 | 2,802 | 3,244 | 1,927 | 2,371 | 2,452 | 4,902 | | |
|  |  | Vaccination Coverage | 80.0% | 69.7% | | 57.2% | 65.6% | 71.4% | 81.0% | 85.4% | 92.3% | 92.6% | 91.6% | | |
|  |  | Nominated Population | 28,028 | 759 | | 2,234 | 3,044 | 3,924 | 4,005 | 2,256 | 2,569 | 2,648 | 5,351 | | |
|  |  | Vaccinated Population | 19,844 | 309 | | 936 | 1,537 | 2,292 | 2,912 | 1,780 | 2,281 | 2,404 | 4,805 | | |
|  |  | Vaccination Coverage | 70.8% | 40.7% | | 41.9% | 50.5% | 58.4% | 72.7% | 78.9% | 88.8% | 90.8% | 89.8% | | |

Each city's data is presented in two rows: the top row represents the first dose, and the bottom row represents the second dose. Although the target population was the number of registered residents, the actual vaccination rates exceeded 100% in some cases. This occurred because vaccinations were administered based on the city of residence, including to residents of elderly care facilities within the city who had not yet transferred their residency cards. Furthermore, as mentioned in the main text, the opt-in data for City B has been excluded from the analysis.
